# Genetically modified foods and human health: a comprehensive review and cross-national time-trend analysis

**DOI:** 10.1080/21645698.2026.2634489

**Published:** 2026-03-02

**Authors:** Ah Young Kim, Bora Lee, Da in Choi, Han Yong Lee

**Affiliations:** a Institute of Well-Aging Medicare & Chosun University G-LAMP Project Group, Chosun University, Gwangju, Republic of Korea; b Institute of Health & Environment, Seoul National University, Seoul, Republic of Korea; c Department of Biology Science, BK21 Four Educational Research Group for Age-Associated Disorder Control Technology, Chosun University, Gwangju, Republic of Korea

**Keywords:** Genetically modified organisms, GM foods, human health, public acceptance

## Abstract

GMOs have remained at the center of scientific and societal debate since their regulatory authorization and subsequent market introduction in the 1990 s. This review synthesizes epidemiological evidence from the literature, including observational studies and prior systematic reviews, alongside international policy frameworks, to evaluate potential associations between GMO exposure and human health. Current evidence does not support consistent causal links between GMO consumption and cancer, reproductive toxicity, allergies, or other chronic diseases. We systematically searched PubMed, Web of Science, and international health databases, applied predefined inclusion and exclusion criteria, and synthesized national-level epidemiological data using time-trend and Joinpoint regression analyses. Across countries and disease categories, no consistent temporal alignment was observed between GMO authorization and changes in disease incidence, and pooled breakpoint analyses showed heterogeneous patterns with estimates frequently overlapping zero. Overall, the findings provide no consistent epidemiological support for associations between GMO consumption and major chronic diseases.

## 1. Introduction

GMOs are defined as living organisms whose characteristics have been modified through artificial manipulation of specific genes. The Flavr Savr tomato, the first GM crop approved for commercial sale in the United States, received product-level approval from the FDA in 1994, marking a milestone in agricultural biotechnology.^1,2^ Subsequently, the rapid development and commercialization of GMOs have had a profound influence on modern agriculture, public health, and biotechnology as a whole, yielding various beneficial outcomes such as increased agricultural productivity, improved resistance to pests and diseases, and environmental protection through reduction of pesticide use.^3–5^ In parallel, the restructuring of existing GMO regulatory frameworks, and discourse regarding social acceptance, have expanded globally. Contemporary GMO technologies primarily involve insertion of specific genetic traits into plants to confer functional characteristics; such methods provide both precision and stability. Nevertheless, regardless of technological advancements, social acceptance of GMOs exhibits substantial regional variation. While countries such as the United States, Brazil, and Canada have pursued commercialization under flexible post-market regulatory systems grounded in scientific safety assessments, the EU has maintained a highly cautious stance on GMO approval and distribution in accordance with the precautionary principle.^6–8^ At present, international organizations including the World Health Organization (WHO),^9^ the FAO,^10^ and CODEX continue to evaluate the safety of GM foods available on the market.^11–13^ Major international public health agencies such as the WHO acknowledge the safety of GMO technologies and state explicitly that “no adverse health effects have been reported from the consumption of GM foods.”^14–16^ In 2016, a comprehensive report by the National Academies of Sciences concluded that “there is no substantiated evidence that genetically modified crops have caused adverse health effects in humans.”^17^ Despite this scientific consensus, public concerns regarding GMOs continue.^18–21^ Most assessments of GMO safety have focused on laboratory-based toxicity tests or nutritional comparisons, whereas empirical evaluations such as analyses of disease prevalence in actual populations remain limited.^22–25^ Accordingly, we aimed to assess the associations between GMO consumption and major diseases (e.g., cancer, allergies) through a meta-analytical and cross-national time-trend approach. We did this by linking the timing of GMO regulatory authorization for commercial distribution in each country with authoritative health statistics from sources such as the WHO, the Global Burden of Disease Study (GBD), the Global Cancer Observatory (GLOBOCAN – Cancer Incidence, Mortality and Prevalence Worldwide), the Centers for Disease Control and Prevention, and the European Centre for Disease Prevention and Control (ECDC). This approach enables assessment of epidemiological signals through epidemiological validation beyond simple toxicity tests, while application of statistical models that account for heterogeneity across studies increase the reliability of these analyses. Accordingly, this review seeks to summarize the current body of biological and epidemiological research on the relationship between GMO consumption and disease, and to conduct a meta-analytical assessment of the association between the timing of GMO regulatory authorization for commercial distribution and disease incidence across countries, thereby evaluating whether public health concerns regarding GMOs are scientifically justified. The following central question will be addressed: *“Is there epidemiological evidence supporting a consistent association between GMO regulatory authorization for commercial distribution and major human diseases, particularly site-specific cancers?”* To this end, we undertake an empirical analysis of GMO safety based on major international reports, peer-reviewed studies, and statistical data, with the aim of narrowing the gap between scientific evidence and public perception.

## 2. Global Status and Characteristics of GMOs

Since their initial commercialization in 1996, genetically modified (GM) crops have expanded rapidly; indeed, within just two decades that have become a central component of global agriculture.^26–28^ By 2018, GMOs were being cultivated in approximately 30 countries, with the United States, Brazil, and Argentina accounting for the majority of the global cultivation area.^29,30^ In Asia, India, China, and Pakistan have emerged as major producers, while in Africa, South Africa has focuses on GM maize and cotton.^31^ By contrast, cultivation in Europe (with the exception of Spain) has been largely restricted due to social and political opposition.^32–35^ As of 2023, the global cultivation area of GMOs exceeded 200 million hectares (Mha), of which the Americas account for approximately 70–80%.^36–38^ The primary GMOs under cultivation are maize, soybean, cotton, and canola, which together account for nearly the entire cultivation area. In terms of traits, herbicide tolerance (HT) and insect resistance (IR) have driven commercialization of GMOs, and such crops comprise the majority of plantings.^27,39,40^ By contrast, although traits such as quality enhancement, stress tolerance, and disease resistance have been developed, such crops are grown only to a limited extent. These characteristics extend beyond merely improving agricultural productivity because they are accompanied by shifts in patterns of herbicide use, as well as ecological and social controversies, necessitating policy responses.^26,40–43^ Ultimately, expansion of GMOs is not confined to specific countries or crop types, but has unfolded as a complex issue encompassing agriculture, the environment, and policy, with different patterns across continents and traits.

### 2.1. Global Status of GMO Cultivation and Importation in 2018

**Figure 1.**
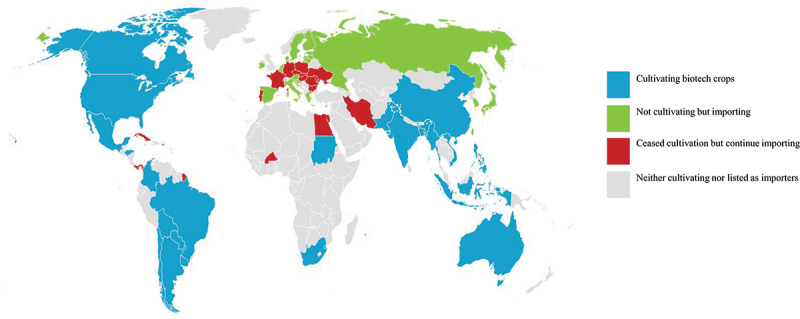
Global distribution of biotech/GM crop adoption in 2018, 23 years after its first regulatory authorization for commercial distribution.

Since the initial commercialization of GMOs in 1996, the global status of adoption and distribution as of 2018 (i.e., 23 years later) has been documented. When we analyzed the cultivation and importation of GMOs by country, we found that national status could be classified into four categories: cultivating countries (Green), non-cultivating importer countries (Blue), countries that discontinued cultivation (Orange), and countries with neither cultivation nor importation (Grey) [Fig. 1]. Cultivating countries comprise approximately 30 nations, primarily distributed across the Americas, Asia, Africa, and parts of Europe and Oceania. Major agricultural exporters in the Americas, such as the United States, Brazil, Argentina, and Canada, were included in this category, with the first three countries accounting for the majority of the global cultivation area. In Asia, India, China, Pakistan, the Philippines, Myanmar, Vietnam, and Bangladesh were classified as cultivating countries, while in Africa, South Africa, Sudan, and Eswatini are included. Australia in Oceania, and Spain in Europe, are also identified as cultivating countries. Non-cultivating importer countries refers to those that did not engage in cultivation but imported GMOs on a large scale. Representative examples are Japan, South Korea, Taiwan, Turkey, and Saudi Arabia in Asia, as well as most member states of the EU. Egypt in Africa, Switzerland in Europe, and New Zealand in Oceania were also categorized as importers. Countries that discontinued cultivation. i.e., those that previously cultivated GMOs but have since halted cultivation while maintaining imports, were concentrated mainly in Europe. These include Germany, France, Portugal, the Czech Republic, Slovakia, Poland, Hungary, Romania, and Bulgaria. Finally, non-cultivating and non-importing countries, where neither cultivation nor importation of GMOs was observed, were mostly located in Africa, as well as parts of Central Asia and the Middle East. Taken together, GMO cultivation as of 2018 was concentrated in approximately 30 countries worldwide, primarily in the Americas. By contrast, many European countries either discontinued cultivation or limited themselves to imports, while most African, Central Asian, and Middle Eastern countries remained relatively inactive on both the cultivation and importation fronts.

Blue (cultivating biotech crops): United States, Brazil, Argentina, Canada, India, China, Paraguay, South Africa, Pakistan, Bolivia, Uruguay, Philippines, Australia, Myanmar, Sudan, Mex ico, Spain, Colombia, Chile, Honduras, Vietnam, Bangladesh, and eSwatini . Green (not cultivating but importing): Japan, South Korea, Taiwan, European Union member states (except Spain [cultivation]), Turkey, Egypt, Saudi Arabia, Switzerland, and New Zealand, others. Red (ceased cultivation but continue importing): Germany, France, Portugal, Cze ch Republic, Slovakia, Poland, Hungary, Romania, Bulgaria. Grey (neither cultivating nor listed as importers): Most countries in Africa, Central Asia, and the Middle Ea st. Classification follows ISAAA (2018, Brief No. 54; GMO Approval Database). Colors indicate legal/market status, not cultivation area or adoption intensity.

### 2.2. National Status Regarding GMO Adoption

Table 1 shows the status of GMO adoption across 30 countries. The year of GMO authorization, and the status as of 2023, are presented alongside basic national information.^44,45^ The year of GMO authorization is categorized as *Cultivation* and *Distribution*: the former refers to the year in which a country first officially approved the cultivation of GMOs, whereas the latter denotes the year in which the importation and distribution of GMOs (for food, feed, or processing) were first permitted.^27^ A dash (“”–) indicates either the absence of an official approval record, or that the respective category is not permitted in the given country. As of 2023, the GMO level split into two categories. The *Cultivation area* (measured in Mha) classifies the scale of GMO cultivation in each country into five categories. “None” indicates the complete absence of cultivation, whereas “Limited” denotes a cultivation area of less than 0.1 Mha. “Small” refers to a cultivation area of at least 0.1 Mha but less than 1.0 Mha, “Medium” corresponds to 1.0 Mha or more but less than 5.0 Mha, and “Large” applies to areas of 5.0 Mha or greater. The *Distribution level* indicates the extent to which each country permits distribution of GMOs. “Fully” denotes complete authorization for distribution across all uses, whereas “Partially” refers to restricted authorization limited to specific purposes or particular GMO events.^28^ By contrast, “Prohibited” signifies a complete ban on GMO distribution. Accordingly, Table 1 provides a comprehensive comparison of the start dates of GMO cultivation, as well as distribution by country. It also shows the scale of the cultivation area as of 2023, and the extent of distribution authorization. When organized by continent, the Americas emerge as the core region of global GMO cultivation.^46^ Since authorizing both cultivation and distribution in 1994, the United States has maintained the world’s largest GMO cultivation area, exceeding 5.0 Mha (Large), while also fully permitting distribution. Similarly, Brazil, Argentina, and Canada authorized cultivation and distribution in the late 1990s, and all have expanded to the Large (≥5.0 Mha) scale. Mexico authorized both cultivation and distribution in 1996, but currently remains on the Small scale (0.1–<1.0 Mha), whereas Peru permitted distribution only in 2011, and does not engage in cultivation. Thus, in the Americas, the United States, Brazil, and Argentina account for the overwhelming majority of the total cultivation area, followed by countries such as Canada and Mexico.^47^ In Asia, the scale of cultivation remains relatively limited, though there is a gradual trend toward expansion. China authorized cultivation in 1997, and is currently Small scale (0.1–<1.0 Mha).^48,49^ India, Japan, and South Korea have not yet approved GMO cultivation; however, the Philippines (2003) and Vietnam (2014) have permitted small-scale cultivation, and both are classified within the Small category. Bangladesh authorized cultivation in 2013, but remains at the Limited level (<0.1 Mha), whereas Pakistan has permitted cultivation since 2005, and has reached the Medium scale (1.0–<5.0 Mha). Although the overall cultivation area in Asia remains limited, the region demonstrates strategic importance for the expansion of GMOs in terms of population size and food demand. In general, Europe has been reluctant to approve GMO cultivation.^50^ Only Spain, which authorized cultiv ation in 1998, maintains small-scale production at the Limited level (<0.1 Mha), while countries such as France, Germany, Italy, Denmark, Sweden, and Norway permit distribution but engage in no cultivation whatsoever. Notably, Switzerland and Serbia prohibit even distribution (*Prohibited*), underscoring that Europe maintains the most conservative policies toward GMOs. In Africa, GMO cultivation has been expanding gradually. South Africa authorized cultivation in 1997, and has since established itself at the Large scale (≥5.0 Mha), whereas Nigeria approved cultivation in 2019, but remains at the Limited level (<0.1 Mha). In most other African countries, however, the authorization of cultivation remains highly limited. In Oceania, Australia has authorized GMO cultivation since 1996, and currently maintains a Medium scale (1.0–<5.0 Mha), whereas New Zealand permits only partial distribution and does not engage in cultivation.^47^ Overall, the United States, Brazil, and Argentina are confirmed as the dominant centers of GMO cultivation, together accounting for 70–80% of global cultivation area. From a policy perspective, Europe maintains the most conservative regulatory stance, while Asia and Africa exhibit a gradual trend toward expansion.

**Table 1.** *National status of GMO adoption: first approval years* for cultivation and distribution, and 2023 cultivation/distribution levels (30 countries).

	Basic information			GMO permitted year		GMO level (2023)	
No	Country	ISO	Continent	Cultivation	Distribution	Cultivation area (Mha)	Distribution level
1	Argentina	ARG	America	1996	1996	Large (≥5.0)	Fully
2	Australia	AUS	Oceania	1996	1996	Medium (1.0 to <5.0)	Fully
3	Bangladesh	BGD	Asia	2013	2013	Limited (<0.1)	Fully
4	Brazil	BRA	America	1998	1995	Large (≥5.0)1	Fully
5	Canada	CAN	America	1995	1995	Large (≥5.0)1	Fully
6	China	CHN	Asia	1997	1997	Small (0.1 to <1.0)	Fully
7	Germany	DEU	Europe	2005	2003	None	Fully
8	Denmark	DNK	Europe	–	2004	None	Partially
9	Spain	ESP	Europe	1998	2004	Limited (<0.1)	Fully
10	Finland	FIN	Europe	–	2004	None	Fully
11	France	FRA	Europe	1997	2004	None	Fully
12	India	IND	Asia	–	2013	None	Partially
13	Italy	ITA	Europe	–	2004	None	Partially
14	Japan	JPN	Asia	–	2001	None	Partially
15	Republic of Korea	KOR	Asia	–	1997	None	Partially
16	Mexico	MEX	America	1996	2005	Small (0.1 to <1.0)	Partially
17	Nigeria	NGA	Africa	2019	2015	Limited (<0.1)	Partially
18	New Zealand	NZL	Oceania	–	2001	None	Partially
19	Pakistan	PAK	Asia	–	2005	Medium (1.0 to <5.0)	Partially
20	Peru	PER	America	–	2011	None	Partially
21	Philippines	PHL	Asia	2003	2003	Small (0.1 to <1.0)	Partially
22	Poland	POL	Europe	–	2004	None	Partially
23	Russian Federation	RUS	Europe	–	1999	None	Partially
24	Sweden	SWE	Europe	–	2004	None	Partially
25	United States of America	USA	America	1994	1994	Large (≥5.0)s	Fully
26	Viet Nam	VNM	Asia	2014	2016	Small (0.1 to <1.0)	Partially
27	South Africa	ZAF	Africa	1997	1997	Large (≥5.0)o	Fully

Country-level GMO adoption status. For each of 30 countries, the first approval year for cultivation and distribution is reported, along with 2023 cultivation area class (None; Limited <0.1 Mha; Small 0.1–<1.0 Mha; Medium 1.0–<5.0 Mha; Large ≥5.0 Mha) and distribution level (Fully/Partially/Prohibited). ISO = ISO-3166 alpha-3 code; Mha = million hectares.

### 2.3. Continental Patterns of GMO Cultivation

When classified by continent [Table 2], the major GMOs are primarily maize, soybean, and cotton, with cultivation areas reaching tens of millions of hectares. Brazil and Argentina, together with the United States, constitute the three leading producers, and have spearheaded expansion of GMOs in South America. Both countries authorized GMO cultivation in the early 2000 s and currently rank among the global leaders in the production of soybean and maize. In North America, Canada has established itself as a major producer, with extensive cultivation focused on canola. In Asia, India has cultivated GMOs, primarily cotton, since 2002, and has since grown into the fourth-largest producer worldwide. China has engaged in limited cultivation centered on *Bt* (*Bacillus thuringiensis*) cotton, but more recently has moved toward expanding other GM maize and soybean production. Pakistan has also increased its GMO acreage rapidly, largely through cotton cultivation. Several other Asian countries, including the Philippines, Myanmar, Vietnam, and Bangladesh, have authorized cultivation of GM maize and rice, thereby accelerating regional adoption. Overall, expansion of GMOs in Asia has concentrated on cotton and maize, with India and China playing leading roles. In Africa, cultivation remains relatively limited, but includes important producers. South Africa was the first country on the continent to adopt GMOs, with cultivation concentrated on maize and cotton. Sudan and Eswatini have likewise expanded adoption, primarily through cotton production. In Oceania, Australia remains the only country to maintain commercial cultivation of GMO, with production centered on canola and cotton. Within Europe, Spain is an exception, permitting commercial cultivation of GM maize and maintaining its role as the principal adopter within the EU.

**Table 2.** Global distribution of GMO crops by continent and country (2023).

Continent	Country	Main GMO Crops	Characteristics
Americas	USA	Corn, Soybean, Cotton	First approval in 1996, world’s largest area
	Brazil	Soybean, Corn	Largest in South America, top 3 globally with USA
	Argentina	Soybean, Corn	Leading GMO expansion in South America with Brazil
	Canada	Canola, Corn, Soybean	Canola-centered, major producer in North America
	Paraguay	Soybean, Corn	Expanding adoption in South America
	Bolivia	Soybean	Limited cultivation allowed
	Uruguay	Soybean, Corn	Export-oriented agriculture
	Colombia	Corn, Cotton	
	Chile	Corn	Mainly for testing and seed production
	Mexico	Corn	Limited cultivation allowed
	Honduras	Corn	Expanding adoption in Central America
Asia	India	Cotton	Approved in 2002, world’s 4th largestn
	China	Cotton (Bt), Corn, Soybean	Limited cultivation, recently expanding
	Pakistan	Cotton	Rapid expansion
	Philippines	Corn	First in Asia to commercialize GMO corn
	Bangladesh	Rice (also Bt Brinjal)	Only Asian country to approve GMO rice
Africa	South Africa	Corn, Cotton	First adopter in Africa
Oceania	Australia	Canola, Cotton	Only country in Oceania with commercial GMO
Europe	Spain	Corn	Only EU country with commercial GMO crops

Major producing countries, primary GMO crops, and notable characteristics are summarized. The Americas dominate (soybean, corn, cotton), Asia is led by India and China (cotton, corn), Africa is limited to few adopters (corn, cotton), Oceania is represented only by Australia (canola, cotton), while in Europe only Spain continues commercial cultivation.

### 2.4. Distribution of Major Trait Characteristics

**Figure 2.**
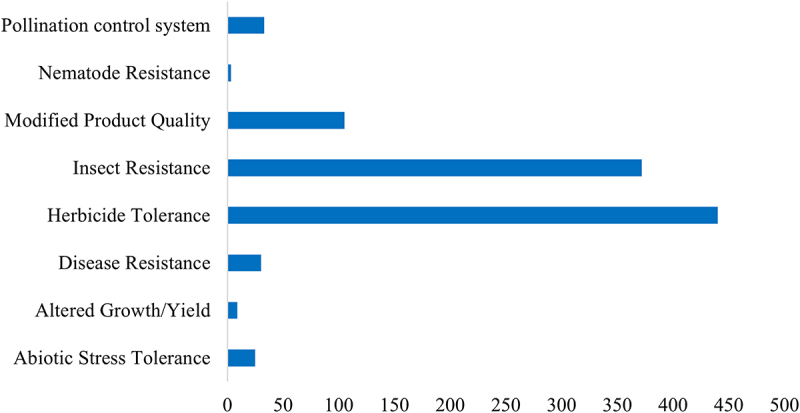
Distribution of biotech crop events by trait category.

When comparing the number of GMO events by trait, herbicide tolerance (HT) and insect resistance (IR) clearly dominate. Based on estimates derived from bar length (axis scale 0–500), HT accounts for approximately 440 events and IR for about 370 events, together totaling roughly 810 events (or about 80% of all cases). Modified product quality (approximately 110 events) follows as the next most common category, while other traits are represented at considerably lower frequencies. Specifically, disease resistance (~30 events), pollination control systems (~25 events), abiotic stress tolerance (~25 events), altered growth/yield (~10 events), and nematode resistance (~5 events) were observed [Fig. 2]. A closer examination reveals three notable characteristics. First, HT and IR account for about four-fifths of all cases, due mainly to the ease of development through production costs incurred during cultivation. Second, output traits related to product characteristics, particularly modified product quality events (on the order of ~100), form a meaningful category, although they remain less prevalent than HT and IR. Third, adoption of traits conferring resistance to biotic and abiotic stresses is limited. Traits such as disease resistance, abiotic stress tolerance, and nematode resistance are restricted to a small number of events. The predominance of HT within the current distribution of GMO events indicates that beyond trait development itself, risk mitigation mechanisms, including residue tolerance standards and monitoring by regulatory authorities, periodic reevaluation of safety, and the incorporation of integrated weed management, are essential.^51^

Horizontal bar graph showing the number of genetically modified (GM) crop events, classified by trait category. The Y axis lists trait categories (i.e., pollination control system, nematode resistance, modified product quality, insect resistance, herbicide tolerance, disease resistance, altered growth/yie ld, abiotic stress tolerance), and the X axis indicates the event count for each category.

## 3. Public Perception and Scientific Evidence on the Health Associations of GMOs

The debate over GMO safety has been shaped not only by scientific data but also by the intersection of agricultural management practices and broader social contexts. Glyphosate resistance genes were used to develop HT, and as using of glyphosate is markedly increased in the 1990 s, the International Agency for Research on Cancer (IARC) has classified this compound as “probably carcinogenic to humans (Group 2A),” which contributed to the widespread public perception that “GMOs = health risks”^52–54^; however, this perception has been amplified more by concerns over pesticide use, debates on carcinogenicity, and media coverage than by direct scientific evidence.^19,20,55–57^ Most studies in the area of disease-related research have not identified significant associations between GMO exposure and adverse health outcomes. Although there is limited evidence for a link to non-Hodgkin lymphoma, multiple myeloma, and certain reproductive outcomes, the observed effect sizes were small, and methodological limitations preclude establishing causality.^58–63^ Likewise, large-scale studies on metabolic, cardiovascular, autoimmune, and allergic diseases have not demonstrated statistically significant associations, and toxicological and allergenicity assessments have failed to substantiate specific health risks. Accordingly, at present, no direct causal relationship between GMOs and specific diseases has been established, and the limited evidence obtained so far appears to be primarily related to herbicide use patterns.^24,64–72^ Future research will require more precise exposure metrics, long-term prospective cohorts, and consideration of multiple pesticide exposures. At the same time, understanding the pathways through which public concerns are shaped remains essential if we are to foster a balanced discourse between science and society.

### 3.1. Pathways Responsible for Public Concern About GMO Safety

**Figure 3.**
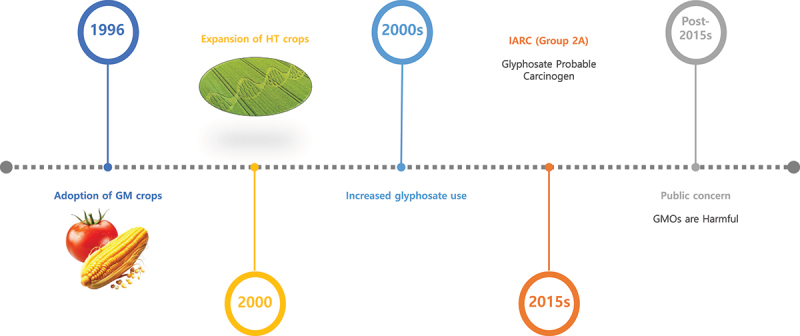
Timeline of GM crop adoption, glyphosate use, and emergence of public concern.

Public concerns regarding GMO safety have been shaped less by the inherent toxicity of the technology itself than by the interplay between changes in agricultural systems and broader social factors. Since the regulatory authorization and subsequent commercial introduction of GMOs in the 1990 s, genetically modified organisms have had a profound impact on modern agriculture, public health discourse, and biotechnology development.^39,73–78^ During this process, anxieties surrounding GMOs have been amplified not so much by scientific data as by external factors such as increased use of pesticides, environmental associations, regulatory uncertainty, and media reporting.^4,52,73,79–86^ As a result, the perception that “GMOs = risk” has become rooted, and is based less on direct toxicological evidence than on deficits of social trust, shortcomings in risk communication, and ongoing institutional controversies [Fig. 3]. This context means that the GMO safety debate extends beyond the scientific evaluation of biotechnology *per se*, and constitutes a complex societal issue that is entangled with agricultural management, chemical regulation, and the processes of social consensus-building.

This timeline illustrates the sequence of key events shaping public perception of GMO safety. GM crops were first adopted in 199 6, followed by the expansion of herbicide tolerant (HT) crops in the early 2000s, which led to increased use of glyphosate. In 2015, the International Agency for Research on Cancer (IARC) under the WHO classified glyphosate as a probable human carcinogen (Group 2A). This classification triggered heightened public concern and the widespr ead perception that GMOs are harmful, despite the fact that the classification referred to glyphosate exposure rather than the genetic modification itself.

### 3.2. Current State of Research on GMO-Disease Associations

To evaluate potential associations between GMOs and specific diseases, we conducted a comprehensive synthesis of the literature by disease category [Table 3]. Across most disease groups, no clear statistical associations with GMOs were identified, and the existing epidemiological evidence was found to be inconsistent or insufficient.^88,89,99,100^ Nevertheless, several studies reported signals at the level of “possible” or “inconclusive” associations.^91,92,94,98,101^ A common feature of these studies was that the exposure metrics were not direct indicators of GMOs themselves, but rather proxies closely linked to the cultivation of HT crops (specifically herbicide use), or regional and dietary surrogates. In addition, effect sizes were generally small, confidence intervals were wide, and heterogeneity was evident across studies in terms of design, adjustment for confounders, and exposure assessment methods.^90,94^ Accordingly, the current level of evidence can be classified as “limited/insufficient,” and caution is warranted in drawing causal inferences. Importantly, these “possible” or “inconclusive” associations largely reflect contexts where HT crop adoption serves as a proxy for increased herbicide use, particularly glyphosate, rather than indicating intrinsic toxicity of GMOs themselves.^87,92,94,98^ This distinction is essential to avoid conflating trait effects with the agronomic management practices that accompany their commercialization. The evidence regarding the intrinsic toxicity of GMOs remains limited.^88,89,99^ While some indicators suggested increased risk, exposure-response relationships were inconsistent, and the effects disappeared in sensitivity analyses.^92,94^ The strengths of these studies included their prospective design and repeated exposure assessments; however, substantial residual confounding remained possible due to policy changes before and after the introduction of HT crops, concurrent use of other pesticides, and environmental factors. Ultimately, these limitations made it difficult to disentangle the potential effects of GMOs themselves from those attributable to herbicide use. By contrast, numerous large-scale cohort and case control studies investigating metabolic disorders, cardiovascular diseases, gastrointestinal conditions, respiratory diseases, autoimmune disorders, and allergic diseases have not reported significant associations with GMOs.^95–97,99^ Furthermore, standard toxicological and allergenicity assessments, including digestive stability tests, protein sequence comparisons, and acute and chronic toxicity studies, have not identified any specific evidence of harm.^88,89,96,99^ In summary, while a limited number of studies suggest possible associations between GMOs and outcomes such as non-Hodgkin lymphoma, multiple myeloma, birth outcomes, and lymphoma, the overall evidence to date remains restricted and insufficient. Across most disease groups, no clear statistical associations with GMOs have been observed, and the few reported signals were more often related to herbicide use than to GMOs themselves. Accordingly, at this stage, it is difficult to assert a direct causal relationship between GMOs *per se* and human disease. Future research will require accurate individual-level exposure metrics, long-term prospective cohorts, and refined analyses that take into account simultaneous exposures to multiple pesticides. Building on the evidence summarized in ‘Public Perception and Scientific Evidence on the Health Associations of GMOs’, the subsequent analyses move from study-level findings to a complementary population-level assessment. As discussed above, the current literature provides no established causal link between GMOs per se and specific diseases, and the limited “possible/inconclusive” signals often depend on proxy indicators that are closely tied to HT-crop adoption and associated herbicide-use patterns rather than direct measures of GMO intake. Moreover, heterogeneity in exposure assessment and confounder control complicates causal interpretation across individual studies. Therefore, in ‘Exclusion Criteria and Analytical Procedures’ we apply national time-series and comparative epidemiological approaches to examine whether disease incidence trends exhibit any consistent temporal concordance with the timing of GMO regulatory authorization for commercial distribution as a policy proxy, while also evaluating whether observed inflection points are more plausibly aligned with non-GMO determinants such as screening expansion, infection control, and healthcare improvements. This framework allows a policy-relevant, hypothesis-generating test of the commonly perceived “GMO adoption → increased disease” narrative without implying causal attribution.

**Table 3.** Summary of disease types and their reported association with GMO exposure.

No	Disease type	Association with GMO	Epidemiological Evidence and Mechanistics	Reference
1	Non-hodgkin lymphoma	Possible	Limited epidemiological evidence supports a potential relationship with glyphosate exposure	^87^
2	Chronic diseases (metabolic, cardiovascular)	No	No epidemiologic evidence linking GMO exposure	^88^
	Toxicity/Carcinogenicity			
3	Tumors or Cancer	Uncertain	No causal relationship identified, but further studies warranted	^22^
4	all cancers combined	No	No significant association; possible link with multiple myeloma not statistically confirmed	^89,90^
	Non-Hodgkin lymphoma, NHL			
	Multiple myeloma			
	Leukemia			
	Prostate cancer			
	Lung cancer			
	Melanoma, non-melanoma			
	Colorectal cancer			
	Liver cancer			
5	Food allergy, hypersensitivity	No	No epidemiologic evidence links GMO foods to specific diseases.	^91^
	Gastrointestinal disturbance			
	Obesity, metabolic disorders			
	Cancer risk			
	Reproductive toxicity			
	Secondary health risks			
6	Non-Hodgkin lymphoma, NHL	No	Weak and inconsistent associations observed for non-Hodgkin lymphoma and multiple myeloma; no causal relationship established between glyphosate exposure and lymphohematopoietic cancers.	^92–94^
	Hodgkin lymphoma, HL			
	Multiple myeloma, MM			
	Leukemia			
	Neoplasms			
7		No	No evidence linking GMOs to reproductive toxicity.	^24,89,95^
	Health & Disease Outcomes		Health risk perception from misinformation rather than evidence.	
	Public Perception & Public Health		No causal relationship identified between GM diets and specific diseases in animal health.	
8	Allergic diseases	No	No epidemiological data supporting allergy risk from GMO foods.	^96,97^
9	Cancer	No	No evidence links GMO exposure to cancer or adverse health effects.	^93^
	Other health effects (liver, kidney, reproductive, endocrine, etc.)			
	public exposure			
10	Non-Hodgkin lymphoma	Possible	Agricultural study shows glyphosate exposure may elevate NHL risk.	^60,98^
	Lymphoma		Meta-analysis suggested potential glyphosate link to NHL.	
11	Hepatic and renal toxicity	No	No scientifically proven causal relationship.	^99^
	Pancreatic and hepatic tissue alterations			
	Reproductive system changes (ovarian and uterine alterations)			
	Gastritis (stomach inflammation)			
	Histopathological changes in the small intestine and testes			
	Genotoxicity (genetic damage)			

This table compiles epidemiological evidence and mechanistic insights from the literature regarding potential links between genetically modified organisms (GMOs) and various diseases. For each disease type, the presence or absence of association with GMO exposure is indicated, along with supporting or contradictory findings from epidemiological and mechanistic studies. The majority of disease categories show no confirmed link with GMO consumption or exposure. A limited number of studies report possible associations, primarily mediated by herbicide use (e.g., glyphosate in herbicide-tolerant crops), but evidence remains inconsistent and often inconclusive.

## Analytical Methods

### Overview of Epidemiological Analyses on GMO Introduction and Disease Trends

In this section, we present the epidemiological analyses used to evaluate potential associations between GMO adoption and trends in specific disease types. We first outline the study design and data sources, including the literature search and selection procedures, exclusion criteria, and key analytical methods based on national classification systems, exposure variables, and disease indicators. We then report the results of Joinpoint regression analyses conducted to detect inflection points in national disease incidence trends in relation to the timing of GMO adoption. In addition, trends in disease incidence for major conditions – particularly liver cancer and non-Hodgkin lymphoma (NHL) – are examined using data from the Global Burden of Disease (GBD). Analyses were restricted to countries with long-term, continuous incidence data and a clearly defined year of GMO authorization, which were required for Joinpoint and time-trend analyses. Countries or disease endpoints with substantial data gaps, inconsistent registry coverage, or insufficient follow-up duration were excluded from specific analyses. Missing data were handled by excluding the corresponding country – endpoint combinations rather than by imputation. This integrated analytical framework provides the basis for the interpretation of the results and for evidence-informed discussions of GMO safety and related policy implications.

### Exclusion Criteria and Analytical Procedures

**Figure 4.**
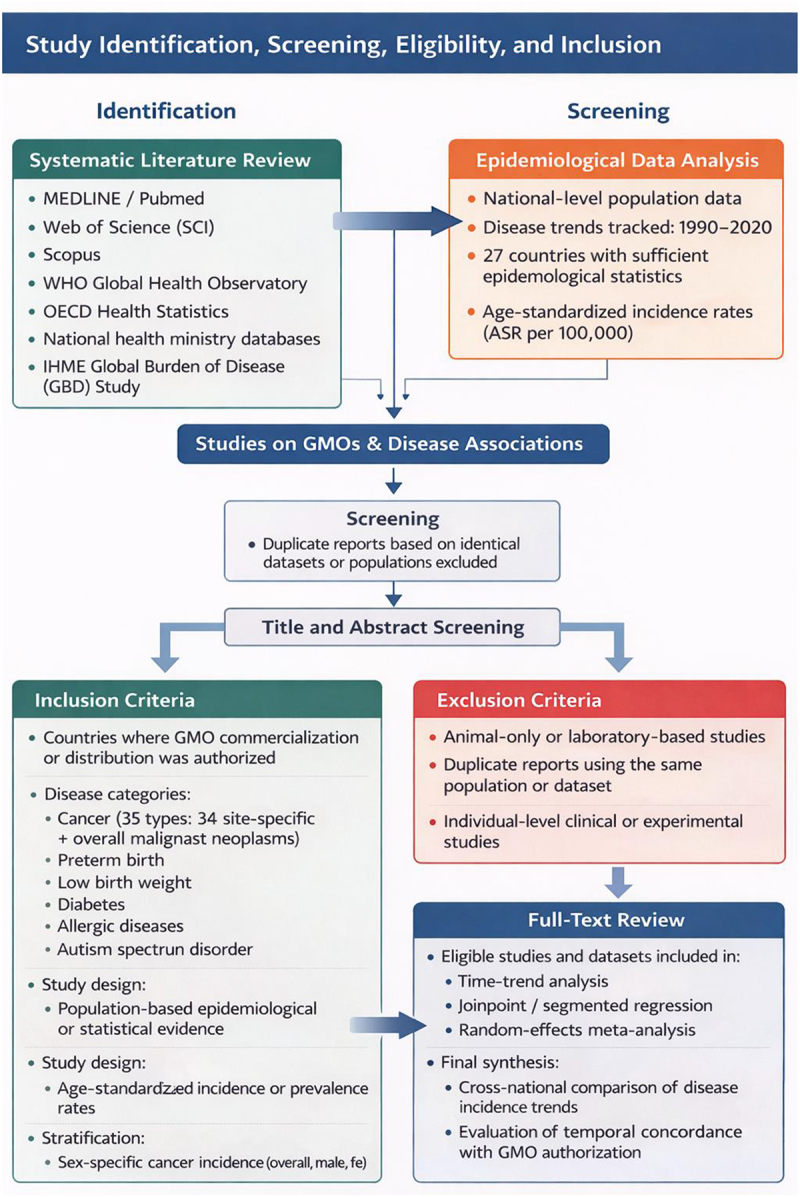
PRISMA flow diagram illustrating the processes of study identification, screening, eligibility assessment, and inclusion.

This study adopted a mixed research design that combined a systematic review of the literature with an analysis of national-level epidemiological data in order to evaluate the associations between GMOs and the incidence of specific diseases. The literature search focused primarily on major academic databases, including PubMed and Web of Science, but also incorporated data from international organizations such as the WHO and the Food and Agriculture Organization of the United Nations (FAO). The search period was restricted to 1996–2023, corresponding to the years following the commercial introduction of GMOs. The inclusion criteria were as follows: (1) epidemiological or health statistics reported from countries where commercial distribution of GMOs had been authorized; (2) disease categories limited to cancer, autism spectrum disorder, diabetes, and allergic diseases; and (3) provision of population-based statistical or epidemiological evidence. The exclusion criteria were: (1) studies based solely on animal experiments; and (2) duplicate reports based on the same study population or dataset. Based on these criteria, duplicate records identified in the initial search were removed, followed by screening of titles and abstracts. Final inclusion was determined through full-text review (Fig. 4). Because this study relied exclusively on published literature and aggregated, non-identifiable secondary data, ethics committee approval was not required. The study was conducted and reported in accordance with the PRISMA 2020 guidelines for systematic reviews.^102^

The diagram summarizes the integrated workflow combining systematic literature review and national level epidemiological data analysis used in this study.

### Study Countries and Classification Criteria

Based on age-standardized incidence rates per 100,000 population (extracted from the GBD database provided by the Institute for Health Metrics and Evaluation (IHME)), we conducted an analysis of potential associations between national GMO authorization status (as a proxy for exposure) and disease incidence. Disease endpoints were defined according to the Global Burden of Disease (GBD) classification, which harmonizes underlying ICD coding across countries and time periods. This approach ensured comparability of incidence estimates across national datasets. The disease categories were restricted to liver cancer, non-Hodgkin lymphoma, leukemia, and multiple myeloma. For the Joinpoint regression analysis, countries were selected according to the level of regulatory authorization for commercial distribution and then classified into two groups. The first group comprised countries that had actively commercialized GMOs, including the United States, Canada, Brazil, and the Philippines. The second group consisted of countries with more restricted GMO adoption, such as member states of the EU, Japan, and South Korea. In total, 30 countries with sufficient disease incidence data were included in the descriptive analyses (Table 1); among these, 27 countries with clearly defined GMO authorization years were eligible for Joinpoint gap estimation and were included in the breakpoint and pooled meta-analyses (Table 4).

**Table 4.** *Gap between GMO approval year* and cancer incidence breakpoints across 27 countries (1990?2020).

	Basic information				Gap between breakpoint of incidence rate and GMO allowed year (95% CI) per cancer type				
No	ISO	Country	Continent	GMO allowed year*	Neoplasms (all cancers)	Stomach	Colon and rectum	Breast (in female)	Liver
1	ARG	Argentina	America	1996	22.4 (22.1, 22.7)	14.9 (14.0, 15.8)	15.6 (14.7, 16.5)	10.7 (9.6, 11.8)	6.4 (6.1, 6.7)
2	AUS	Australia	Oceania	1996	8.2 (7.1, 9.3)	8.8 (7.8, 9.8)	8.2 (7.1, 9.3)	−1.2 (−2.0, −0.4)	20.5 (20.2, 20.8)
3	BGD	Bangladesh	Asia	2013	1.8 (0.4, 3.2)	−4.1 (−5.6, −2.6)	−4.1 (−5.3, −2.9)	−12.4 (−13.3, −11.5)	−3.3 (−3.9, −2.7)
4	BRA	Brazil	America	1995	4.3 (3.5, 5.1)	5.0 (4.5, 5.5)	24.4 (23.1, 25.7)	−0.4 (−1.9, 1.1)	16.2 (15.5, 16.9)
5	CAN	Canada	America	1995	1.8 (0.7, 2.9)	12.7 (11.4, 14.0)	13.7 (13.1, 14.3)	1.4 (0.2, 2.6)	20.5 (20.0, 21.0)
6	CHN	China	Asia	1997	21.7 (21.4, 22.0)	6.8 (6.1, 7.5)	2.4 (1.2, 3.6)	18.3 (17.9, 18.7)	17.7 (17.5, 17.9)
7	DEU	Germany	Europe	2003	11.7 (11.1, 12.3)	−6.2 (−6.9, −5.5)	−5.8 (−6.3, −5.3)	−6.3 (−6.8, −5.8)	−7.7 (−8.5, −6.9)
8	DNK	Denmark	Europe	2004	−7.6 (−8.3, −6.9)	8.9 (8.3, 9.5)	−7.3 (−7.9, −6.7)	4.7 (4.0, 5.4)	4.4 (3.7, 5.1)
9	ESP	Spain	Europe	2004	−7.6 (−8.3, −6.9)	−1.9 (−2.7, −1.1)	8.1 (6.2, 10.0)	−8.4 (−9.2, −7.6)	1.4 (0.4, 2.4)
10	FIN	Finland	Europe	2004	−6.5 (−7.1, −5.9)	−3.9 (−4.4, −3.4)	4.0 (2.3, 5.7)	−8.0 (−11.8, −4.2)	2.6 (0.5, 4.7)
11	FRA	France	Europe	2004	15.3 (14.5, 16.1)	3.7 (2.7, 4.7)	6.3 (4.7, 7.9)	5.5 (4.4, 6.6)	7.3 (6.3, 8.3)
12	IND	India	Asia	2013	−8.0 (−9.9, −6.1)	−1.7 (−3.2, −0.2)	−16.5 (−17.5, −15.5)	3.7 (3.3, 4.1)	−7.5 (−9.0, −6.0)
13	ITA	Italy	Europe	2004	5.5 (4.5, 6.5)	7.3 (6.0, 8.6)	1.3 (−0.6, 3.2)	−8.1 (−11.8, −4.4)	15.4 (13.9, 16.9)
14	JPN	Japan	Asia	2001	3.3 (2.8, 3.8)	1.0 (−0.3, 2.3)	0.7 (−0.4, 1.8)	1.4 (0.4, 2.4)	9.3 (7.8, 10.8)
15	KOR	Republic of Korea	Asia	1997	19.4 (18.3, 20.5)	0.8 (0.1, 1.5)	18.7 (17.9, 19.5)	18.6 (17.3, 19.9)	13.5 (13.1, 13.9)
16	MEX	Mexico	America	2005	0.9 (0.1, 1.7)	7.0 (5.9, 8.1)	6.3 (5.5, 7.1)	−7.0 (−8.3, −5.7)	−2.7 (−4.4, −1.0)
17	NGA	Nigeria	Africa	2015	−21.4 (−22.1, −20.7)	−21.3 (−22.0, −20.6)	−1.7 (−3.1, −0.3)	−21.6 (−22.3, −20.9)	−8.5 (−9.1, −7.9)
18	NZL	New Zealand	Oceania	2001	2.0 (0.8, 3.2)	−7.0 (−8.8, −5.2)	−3.1 (−3.9, −2.3)	1.3 (0.1, 2.5)	−1.4 (−1.8, −1.0)
19	PAK	Pakistan	Asia	2005	−11.0 (−12.4, −9.6)	−1.8 (−2.7, −0.9)	−1.7 (−2.8, −0.6)	−10.9 (−12.2, −9.6)	0.2 (−1.0, 1.4)
20	PER	Peru	America	2011	4.6 (2.0, 7.2)	−6.0 (−12.6, 0.6)	4.5 (1.7, 7.3)	−5.0 (−10.9, 0.9)	4.6 (3.7, 5.5)
21	PHL	Philippines	Asia	2003	1.4 (−0.3, 3.1)	−1.9 (−3.6, −0.2)	4.0 (1.3, 6.7)	10.3 (10.1, 10.5)	8.3 (7.8, 8.8)
22	POL	Poland	Europe	2004	0.4 (−0.5, 1.3)	5.2 (0.1, 10.3)	−0.5 (−2.1, 1.1)	−4.2 (−4.6, −3.8)	0.5 (−0.9, 1.9)
23	RUS	Russian Federation	Europe	1999	8.0 (6.7, 9.3)	−1.2 (−1.9, −0.5)	−1.3 (−2.1, −0.5)	8.0 (6.1, 9.9)	2.1 (1.0, 3.2)
24	SWE	Sweden	Europe	2004	14.8 (14.1, 15.5)	−9.2 (−11.4, −7.0)	14.9 (14.1, 15.7)	−7.7 (−8.7, −6.7)	−8.8 (−9.5, −8.1)
25	USA	United States Of America	America	1994	12.4 (12.0, 12.8)	7.9 (6.1, 9.7)	6.6 (5.8, 7.4)	1.2 (0.7, 1.7)	12.0 (11.3, 12.7)
26	VNM	Viet Nam	Asia	2016	−6.9 (−7.9, −5.9)	−6.5 (−7.1, −5.9)	−14.3 (−15.8, −12.8)	−8.3 (−9.1, −7.5)	−9.8 (−11.0, −8.6)
27	ZAF	South Africa	Africa	1997	7.7 (6.5, 8.9)	1.0 (−0.4, 2.4)	18.5 (17.2, 19.8)	2.6 (1.3, 3.9)	4.4 (3.7, 5.1)
Pooled estimates for gap between breakpoint year of incidence rate and GMO allowed year**		Total (27)			3.7 (−0.3, 7.6)	0.7 (−2.2, 3.6)	3.8 (0.1, 7.5)	−0.8 (−4.3, 2.7)	4.4 (0.9, 7.8)
		Per continent		Africa (2)	−6.9 (−35.4, 21.7)	−10.2 (−32.0, 11.7)	8.4 (−11.4, 28.2)	−9.5 (−33.2, 14.2)	−2.1 (−14.7, 10.6)
				America (6)	7.8 (1.1, 14.4)	7.4 (2.1, 12.7)	11.9 (5.8, 17.9)	0.3 (−4.7, 5.3)	9.5 (2.8, 16.2)
				Asia (8)	2.7 (−5.7, 11.2)	−0.9 (−3.7, 1.9)	−1.4 (−9.0, 6.3)	2.6 (−6.1, 11.2)	3.6 (−3.4, 10.6)
				Europe (9)	3.8 (−2.4, 9.9)	0.2 (−3.9, 4.4)	2.2 (−2.4, 6.8)	−2.7 (−7.1, 1.8)	1.9 (−2.9, 6.7)
				Oceania (2)	5.1 (−1.0, 11.2)	0.9 (−14.6, 16.4)	2.5 (−8.5, 13.6)	0.0 (−2.4, 2.5)	9.6 (−11.9, 31.0)
		Per GMO allowed year		<2005 (20)	6.9 (3.0, 10.9)	2.6 (−0.3, 5.5)	6.5 (2.6, 10.3)	2.0 (−1.5, 5.6)	7.2 (3.5, 11.0)
				2005 to 2014 (5)	−2.4 (−8.3, 3.5)	−1.0 (−5.4, 3.4)	−2.3 (−10.2, 5.6)	−6.4 (−12.1, −0.6)	−1.7 (−5.7, 2.2)
				≥2015 (2)7	−14.2 (−28.4, 0.1)	−13.9 (−28.4, 0.6)	−8.0 (−20.3, 4.4)	−15.0 (−28.0, −1.9)	−9.0 (−10.3, −7.8)

This table presents the estimated time gap (years, 95% CI) between the year of GMO approval and the joinpoint year of incidence rate change for four major cancer sites (stomach, colon and rectum, female breast, and liver), as well as all neoplasms combined. Estimates were derived using Joinpoint regression adjusted for demographic and lifestyle covariates (population size, aging ratio, GDP, processed food consumption, daily caloric intake, and smoking prevalence). Positive values indicate that the incidence breakpoint occurred after GMO approval, while negative values indicate that the breakpoint preceded GMO approval. Pooled meta-estimates are shown by continent and by GMO approval period (≤2005, 2005–2014, ≥2015). Results demonstrate heterogeneity across countries and cancer types, with no consistent temporal alignment between GMO adoption and cancer incidence breakpoints. GMO, genetically modified organism; ISO, International Organization for Standardization.

*GMO allowed year for distribution.

**Pooled estimates are calculated based on random effects meta-analysis using inverse-variance weighting(IVW) and variance estimation via restricted maximum likelihood (REML). Numbers in parentheses indicate the number of countries included in each subgroup (per continent and per GMO-allowed year category).

### Definition of Exposure Variables

As a proxy indicator for GMO exposure, the year of authorization for commercial distribution was used as the reference point. Because the approval year for GMO cultivation varies substantially by country, crop, and individual GMO event, this study adopted the onset of large-scale commercial distribution as a unified and policy-relevant criterion. Long-term trends spanning 10–20 years before and after GMO authorization in each country were compared to account for the potential latency of health effects. For subsequent breakpoint analyses, the temporal gap was defined as the difference between the year of cancer incidence breakpoint and the year of GMO authorization (“breakpoint year − authorization year”). Positive gap values indicate that the breakpoint occurred after GMO authorization, whereas negative gap values indicate that the breakpoint preceded authorization.

### Definition of Disease Indicators and Data Collection

The primary diseases included 35 cancer types (34 site-specific cancers plus overall malignant neoplasms Table S1). In addition, conditions of high public concern, such as preterm birth, low birth weight, allergy prevalence, diabetes, and autism spectrum disorder, were also reviewed at the screening stage. Cancer incidence was further stratified by sex (overall, male, and female) where data availability permitted. For the national-level epidemiological analyses, disease incidence data were obtained primarily from the WHO Global Health Observatory,^103^ OECD Health Statistics,^104^ and official national health ministry statistics, supplemented by the Global Burden of Disease (GBD) database provided by the Institute for Health Metrics and Evaluation (IHME).^105^ GBD data are reported as age-standardized incidence rates (ASR) per 100,000 population, ensuring comparability across countries and time periods. While a broad range of disease outcomes was initially considered, the main comparative time-trend analyses presented in the Results section focus on four major cancer outcomes (liver cancer, non-Hodgkin lymphoma, leukemia, and multiple myeloma), for which long-term, continuous GBD incidence data and clearly defined authorization years were available across multiple countries. Incidence rates were examined in relation to the year of GMO authorization in each country, with particular attention to trends observed more than 20 years after the onset of commercial GMO authorization. The analytical time series spanned from 1990 to 2020 to capture long-term population-level patterns.

### Statistical Analyses (Identification of Breakpoints Associated with GMO Adoption)

The statistical analyses in this study were performed in a stepwise manner to quantitatively evaluate the temporal relationship between the timing of GMO authorization and inflection points in disease incidence. As a preliminary trend analysis, time-series evaluations were conducted to compare changes in disease indicators over the 10–20 years before and after the year of GMO authorization. To further assess the temporal relationship between GMO adoption and shifts in disease incidence, we performed Joinpoint regression analyses. Joinpoint regression is a statistical technique that identifies significant changes in trends within continuous time-series data by allowing for inflection points in otherwise linear regression models, thereby enabling more accurate characterization of temporal trends.^106^ This methodology was developed by the National Cancer Institute, and is applied widely for analysis of temporal changes in cancer incidence and mortality rates.^107^ To ensure analytical accuracy, a multiple regression model was first constructed for each country-cancer type-sex combination, adjusting for covariates such as population size, aging rate, GDP, smoking prevalence, caloric intake, and consumption of ultra-processed foods. Subsequently, a single-breakpoint model, using year as the partitioning variable, was applied to estimate structural changes in disease incidence, with the statistical significance of each breakpoint evaluated together with its standard error. The temporal gap between the year of GMO authorization and the year of incidence breakpoint was defined as the difference between these two points. Gap values approaching zero were interpreted as approximate concordance between the breakpoint and the timing of GMO adoption. Had GMOs exerted a substantive effect on disease incidence, consistent directional breakpoints would have been expected across countries within a defined period following GMO authorization. To test this hypothesis, the country-specific gap values were pooled across countries using a random-effects model to account for between-country heterogeneity. Between-study heterogeneity was accounted for by applying the restricted maximum likelihood (REML) method to estimate the between-study variance (τ^2^) and to derive pooled effect estimates. Study weights were calculated as the inverse of the total variance, defined as the sum of within-study variance and the estimated between-study variance. In addition, stratified analyses were conducted by continent (Americas, Europe, Asia, and Oceania) and by period of GMO authorization (pre-2005, 2005–2014, and post-2015) to disentangle the effect of regional and temporal influences on the results. Between-country heterogeneity was assessed using the I^2^ statistic (I^2^ < 25%: low heterogeneity; 25–50%: moderate heterogeneity; 50–75%: substantial heterogeneity; I^2^ > 75%: considerable heterogeneity).^108^ To further quantify the uncertainty of the results, both 95% confidence intervals and prediction intervals were reported. All statistical analyses were conducted using R Statistical Software (v4.4.2; R Core Team, 2024). For Joinpoint regression, the *segmented* package (version 2.1–4) ^109^ was employed, while meta-analyses utilized the *meta* (version 8.1–0) ^110^ and *metafor* (version 4.8–0) ^111^ packages. Statistical significance was determined using two-sided tests with a significance threshold of 0.05.

## 5. Results

### Results of the GBD Analysis

#### Comparative Analysis of Trends in Liver Cancer Incidence and the Timing of GMO Adoption

**Figure 5.**
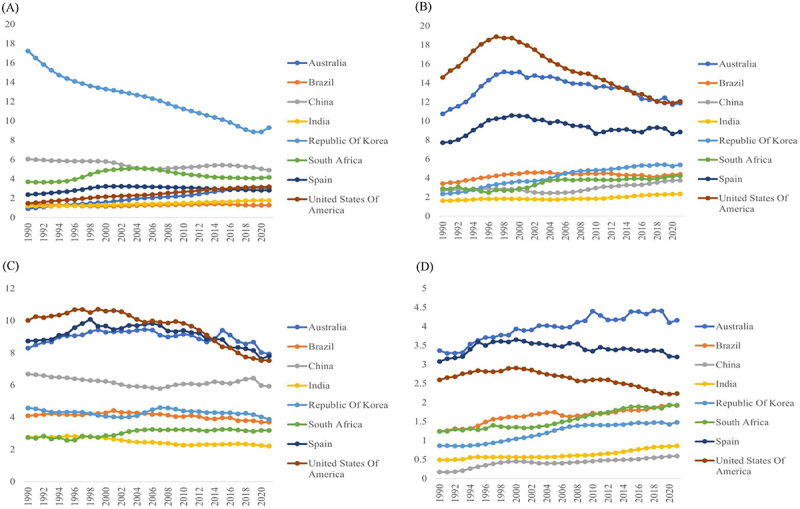
Age standardized incidence trends of selected cancers (1990 2020) in GMO adopting and non adopting countries.

There was a marked difference in liver cancer incidence across countries; however, no direct association with the timing of GMO adoption was identified [Fig. 5(A)]. In South Korea, the ASR in the early 1990s was approximately 18, the highest among the study countries, but declined steadily thereafter, reaching around nine by 2020; this represents a reduction of nearly 45–50%. This pronounced decrease occurred independently of the period during which GMO imports were authorized (late 1990 s to early 2000 s), and is instead attributable to public health interventions such as expanded hepatitis B vaccinations and the wider availability of antiviral treatments (e.g., national HBV control programs and treatment scale-up). In China, the ASR remained at approximately 6, showing long-term stability or only a modest decline, with no discernible change following introduction of *Bt* cotton and authorization of GMO imports in the late 1990s to early 2000s. In South Africa, incidence fell gradually from 4–5 to the high 3 range, without notable shifts after the regulatory authorization for commercial distribution of GM maize in 1998. The United States, Australia, Spain, and Brazil all demonstrated low to moderately low levels (2.5–4), maintaining either a slight decline or as steady trend over the past 30 years. Even after more than 20 years of GMO regulatory authorization for commercial distribution, the United States (1996), Brazil and Spain (early 2000s), and Australia (early 2000s) showed no evidence of increasing liver cancer incidence. In India, the ASR remained stable at the lowest level (1.5–2), with no notable change following regulatory authorization for commercial distribution of *Bt* cotton in 2002. In summary, across all eight study countries, no cases were observed in which liver cancer incidence increased in conjunction with GMO adoption, even after more than 20 years of follow-up. Notably, South Korea exhibited a sharp decline in incidence, irrespective of the timing of GMO import expansion, underscoring the central role of infection control and medical interventions. Other countries likewise maintained low-level incidence, with gradual decreases or stable levels, providing no scientific or statistical support for a temporal or epidemiological correlation between GMO adoption and incidence of liver cancer.

Age standardized incidence rates (per 100,000) for liver cancer (A), non Hodgkin lymphoma (B), leukemia (C), and multiple myeloma a (D) across eight countries (United States of America, Brazil, India, China, Republic of Korea, South Africa, Spain, and Australia). Data are derived from the Gl oba l Burden of Disease (GBD) study.

#### Comparative Analysis of the Incidence of NHL and the Timing of GMO Adoption

The incidence of NHL varied across countries [Fig. 5(B)]; however, we observed no direct correlation with the timing of GMO adoption. The incidence rate in the United States peaked at approximately 18–19 per 100,000 in the mid-1990 s, and then declined steadily, reaching 12–13 per 100,000 by the 2010s. Australia likewise showed a continuous increase through the 1990 s, rising to about 15, but this was followed by a gradual decline in the 2010s, stabilizing at around 13 in recent years. Spain followed a downward trajectory, decreasing from its peak of ~ 11 to about 9. By contrast, emerging economies such as China, South Korea, Brazil, South Africa, and India began at relatively low levels (2–4), but have shown gradual increases in recent years, reaching 3–6. Nonetheless, these increases varied in pace and magnitude across countries, and can generally be summarized as “progressive rises from low baseline levels.” Importantly, these national patterns did not coincide with the timing of GMO regulatory authorization for commercial distribution. For instance, although the United States (1996), Australia (early 2000 s), and Spain (1998) were early adopters of GMOs, NHL incidence did not increase alongside GMO expansion; rather, it declined after the 2000 s. Conversely, in China, India, South Africa, and Brazil, incidence rose modestly after GMO adoption; however, such increases are likely attributable to epidemiological and healthcare-related factors, including delayed control of infections (e.g., AIDS treatment, eradication of Helicobacter pylori), improvements in diagnostic and cancer registration systems, and population aging. In summary, more than 20 years after GMO adoption, no temporal or epidemiological correlation was identified between GMO diffusion and NHL incidence. Divergent patterns were observed, with declines following a peak in high-income countries, and gradual increases in emerging economies, but these trends are more consistently explained by national healthcare systems, management of infectious risk factors, and lifestyle determinants rather than by GMO adoption itself. Accordingly, the available evidence does not support a consistent epidemiological association between GMOs and incidence of NHL.

#### Comparative Analysis of Trends in Leukemia Incidence and the Timing of GMO Adoption

The incidence of leukemia demonstrated either a gradual decline or a stable trend, with no direct association with the timing of GMO adoption. In the United States and Australia, incidence peaked at approximately 10–10.5 in the late 1990s but then declined to 7.5–8 by 2020 [Fig. 5(C)]. In China, incidence began at around 6.5, decreased, and then stabilized, resulting in a pattern resembling a “U-shaped stagnation.” South Korea and Brazil started at moderately low levels (~4.5), which then fell gradually to the high 3 range by 2020. Spain and South Africa remained at relatively low levels (2.5–3), exhibiting stability or a slight decline, while India showed a gradual downward trend from a low baseline (~3). When contrasted with the timing of GMO regulatory authorization for commercial distribution, there were no consistent increases in leukemia incidence in countries for which more than 20 years have elapsed since GMO adoption, i.e., the United States (1996), Spain (1998), Brazil and Australia (early 2000 s), and South Korea (late 1990 s to early 2000 s). On the contrary, the incidence in the United States and Australia declined markedly following GMO regulatory authorization for commercial distribution, whereas in other countries the rates remained low (either stable or a gradual decline). In summary, although there are some minor differences across countries, overall trends in leukemia incidence displayed either a gradual decline or remained stable. These patterns are more plausibly explained by improvements in healthcare systems, advances in diagnostic and therapeutic technologies, and management of environmental and lifestyle factors. Therefore, we identified no temporal or epidemiological correlation between GMO adoption and leukemia incidence, and there was no consistent association.

#### Comparative Analysis of Multiple Myeloma Incidence Trends and the Timing of GMO Adoption

The incidence of multiple myeloma exhibited heterogeneous patterns across countries; however, no direct association with the timing of GMO adoption was identified [Fig. 5(D)]. In Australia, incidence increased steadily from approximately 3 in the early 1990s to 4.3, but has plateaued recently. In the United States, rates declined gradually from 3.1 at baseline to about 2.2 by 2020, while Spain also showed a slight decrease from 3.5 to 3.2. By contrast, South Korea, Brazil, South Africa, China, and India all fell within the low-incidence group, beginning at 0.8–1.0, but rising slowly to 1.3–1.8 by 2020. These increases were generally modest, and the absolute incidence across countries remained comparatively low. When compared with the timing of GMO regulatory authorization for commercial distribution, no consistent upward trend was observed. Specifically, in the two or more decades since authorization of GMO, the United States (1996), Spain (1998), Australia, Brazil, South Korea, China, South Africa (early 2000s), and India (2002) showed no increase in the incidence rates of multiple myeloma. On the contrary, incidence in the United States and Spain fell, whereas Australia experienced an increase followed by stabilization. The gradual increases observed in low-incidence countries are more likely attributable to epidemiological and healthcare-related factors such as population aging, improved diagnostic sensitivity, and enhancements in cancer registry systems, rather than to GMO diffusion. In summary, multiple myeloma demonstrated divergent patterns: modest increases in low- and middle-incidence countries, declines in the United States and Spain, and an increase followed by stabilization in Australia; however, there is no temporal or epidemiological evidence supporting a direct association with GMO adoption. Thus, even after more than 20 years since commercialization, changes in the incidence of multiple myeloma cannot be interpreted as being attributable to GMOs.

### Breakpoint Years of Cancer Incidence Trends and GMO Approval Years by Country

The estimated gaps between the year of GMO authorization/distribution and the year of cancer incidence breakpoint (Joinpoint) are summarized in Table 4. Breakpoints were calculated using adjusted Joinpoint regression models that incorporated the covariates mentioned in the previous section. The resulting country-specific gap estimates were then pooled across 27 countries using random-effects meta-analysis (REML) to generate summary estimates under three stratifications: (1) overall, (2) by continent, and (3) by timing of GMO authorization (pre-2005, 2005–2014, post-2015). The results showed that both the direction and magnitude of the country-specific gap values were inconsistent. Even for the same cancer type, some countries exhibited breakpoints several years-to-decades after GMO authorization, whereas in others the breakpoints had already appeared prior to authorization. This indicates a lack of temporal synchrony or consistency between the timing of GMO approval and occurrence of cancer incidence breakpoints. Moreover, the pooled estimates from the meta-analysis were generally small, of the order of only a few years, while confidence intervals frequently included zero; in addition, substantial between-country heterogeneity was observed, underscoring the need for caution in interpreting the results. For liver cancer, the pooled gap was +1.8 years (95% CI: −2.4 to 6.0; I^2^ = 78%), indicating substantial heterogeneity and no consistent temporal alignment. For certain site-specific cancers (e.g., colorectal cancer, liver cancer), slight positive gaps were detected, suggesting a tendency for breakpoints to occur somewhat more frequently several years after GMO authorization; however, the direction of these breakpoints (increasing *vs*. decreasing) varied across countries. In particular, in high-income countries, the observed downward inflections largely corresponded to non-GMO factors such as the expansion of colorectal cancer screening programs and improved control of hepatitis B virus. Similarly, continental analyses revealed no consistent directional patterns. Although small positive gaps were frequently observed in the Americas, Europe, Asia, and Oceania, the wide ranges and overlaps indicated the absence of any synchronous trends at the continental level. Furthermore, when countries were stratified by timing of GMO authorization (i.e., early adopters, intermediate adopters, and recent adopters), no consistent tendencies were identified. In early-adopting countries (≤2005), breakpoints generally exhibited small positive gaps, indicating a tendency to occur several years after the year of GMO authorization. By contrast, in more recent adopters (≥2015) the gaps were close to zero or negative, reflecting instances in which breakpoints had already emerged prior to authorization. This finding does not support the hypothesis that “common breakpoints would cluster within a defined period around the year of authorization.” If GMOs had a significant influence on cancer incidence, one would expect synchronous and directionally consistent (predominantly increasing) breakpoints to appear across countries within a narrow window around the authorization year (e.g., ±5 years); however, in the present analysis, breakpoint years were widely scattered in relation to GMO authorization, the signs of the gaps were mixed (positive and negative), and the directions of the breakpoints (upward *vs*. downward) varied across countries. When considered in an epidemiological context, the observed breakpoints aligned more closely with non-GMO explanatory factors. For colorectal cancer, downward breakpoints in high-income countries were concentrated in the 2000s, coinciding with the widespread introduction of endoscopic screening. For liver cancer, early downward shifts in East Asia corresponded with the expansion of hepatitis B vaccination and dissemination of antiviral therapies. For breast cancer, improvements in screening and therapeutic accessibility served as major turning points, while overall cancer incidence was more closely associated with health system factors such as declining smoking prevalence and advances in treatment technologies. Accordingly, the observed breakpoints are more plausibly interpreted as aligning with the timing of these non-GMO public health policies and medical interventions rather than with GMO adoption. Accordingly, even if a statistically significant positive gap is observed for a given cancer type, for which the corresponding breakpoint reflects a transition toward decreasing incidence, this would in fact contradict rather than support the hypothesis of GMO-related harm. Moreover, despite applying adjusted Joinpoint regression models that control for demographic structure, age distribution, GDP, processed food consumption, caloric intake, and smoking prevalence, substantial differences in breakpoint patterns across countries was observed. This indicates that such changes cannot be explained adequately by GMOs alone as a single global factor, but are more likely attributable to country-specific determinants such as public health policies, lifestyle factors, and infectious exposure. Meta-analyses stratified by timing of GMO authorization revealed no consistent patterns between early- and late-adopting countries, thereby weakening the hypothesis that “GMO authorization itself triggers cancer incidence breakpoints.” Indeed, the adjusted Joinpoint meta-analysis conducted across 27 countries and four cancer types revealed no systematic temporal alignment between the year of GMO authorization and occurrence of cancer incidence breakpoints. According to the meta-analysis results summarized in Table 4, there is no consistent temporal association between the timing of GMO authorization and occurrence of cancer incidence breakpoints. Gap values varied widely across countries, with positive, negative, and near-zero estimates, thereby ruling out systematic alignment between authorization years and incidence trends. These patterns are further illustrated in Fig. 5, which presents GBD-derived incidence trajectories for major cancers across GMO-adopting and non-adopting countries. As shown, the incidence curves differ substantially between countries, with breakpoints emerging at varying times and in divergent directions. Taken together, the findings presented in Table 4 and Figure 5 confirm that observed changes in cancer incidence are more likely attributable to country-specific determinants such as infection control, screening programs, and lifestyle shifts, rather than to GMO adoption. For certain site-specific cancers, breakpoints were observed more frequently several years after GMO authorization; however, their direction and magnitude varied across countries and, in most cases, corresponded more closely with the timing of non-GMO public health interventions such as the introduction of screening programs, dissemination of vaccines and treatments, and declines in smoking prevalence. Therefore, the simple hypothesis that “GMO adoption leads to synchronous and consistent increases in cancer incidence” is not supported by these data. Furthermore, the status of GMO “authorization” does not directly reflect actual consumption levels or human exposure, which may vary across countries depending on contextual factors such as labeling policies, import structures, and dietary practices. Overall, the adjusted Joinpoint meta-analysis demonstrated no temporal or epidemiological concordance between GMO authorization years and cancer incidence breakpoints.

## 6. Conclusions

The results of this random-effects pooling (meta-analytic synthesis) and structural review suggest no direct causal relationship between GMOs and specific diseases, and that the limited evidence that we do have is more likely attributable to changes in patterns of herbicide use rather than to GMOs themselves. For example, increases in the reported risk for NHL and certain reproductive health indicators appear to be more closely linked with increased use of glyphosate. These findings are consistent with previous evaluations by international bodies such as the WHO, the EFSA, and the U.S. National Academy of Sciences, all of which concluded that scientific evidence remains insufficient to support significant associations between GMO consumption and outcomes such as cancer, allergy, or reproductive toxicity. While some epidemiological studies reported increased risks, the effect sizes were small, confidence intervals wide, and limitations existed in study design and exposure assessment. Toxicity signals reported in animal studies have often not been replicated in humans, and reliance on self-reported dietary data, ecological designs, and residual confounding further reduce reliability. These limitations align with prior meta-analyses and the consensus of international agencies concluding that there is no sufficient epidemiological evidence to substantiate a direct causal link between GMOs and specific diseases. From a societal perspective, public concerns about GMOs are rooted less in scientific evidence than in psychological and social factors. Research indicates that risk perceptions of GMOs are more strongly influenced by media coverage, amplification of fears through social media, and cognitive biases than by scientific knowledge. Even though time-series analyses conducted in the United States, the EU, and the Philippines found no direct associations between GMO authorization and indicators such as cancer, preterm birth, or allergy, public anxiety persists. This disconnect reinforces the perception that “GMO = risk,” and illustrates the gap between scientific consensus and public opinion. At the policy level, and in addition to evidence-based risk assessment, greater transparency and improvements in labeling systems are necessary. Although the EFSA and the FDA apply the same safety standards to GMO foods as conventional foods when there are no significant differences in composition, nutrition, or toxicity (based on the principle of “substantial equivalence”), a binary “contains GMO” label may exacerbate public concerns. Instead, labeling systems should be improved to emphasize whether products have passed safety assessments, as well as providing functional information.

Future research should prioritize: (1) precise, biomarker-based individual-level exposure assessments; (2) long-term cohort studies; (3) analyses that account for mixed pesticide exposures; and (4) advanced statistical techniques, such as Joinpoint regression, for trend detection. In particular, studies comparing breakpoints between the timing of GMO introduction and disease incidence trends may offer valuable epidemiological and policy insights. Moreover, GMO technologies extend beyond safety debates; indeed, GMOs may have the potential to contribute to addressing global challenges such as climate change, food security, and nutritional enhancement. Examples include Golden Rice, drought-tolerant maize, and nitrogen-fixing cereals, which have the potential to improve nutrition and agricultural sustainability. Next-generation genome editing technologies such as CRISPR-Cas9 further raise the need to reconsider existing regulatory frameworks and social acceptance paradigms. Thus, future policy approaches should move beyond the simple dichotomy of “GMO versus non-GMO” to adopt a balanced framework that integrates actual risk, toxicity, and allergenicity with agricultural management systems and broader environmental and health associations. International organizations such as the WHO, FAO, OECD, and Codex Alimentarius have already established international standards for GMO safety and biosafety governance, including the Cartagena Protocol. Moving forward, regulatory harmonization, technology transfer, and the establishment of international standards for trade will be key strategies for ensuring the safe and equitable dissemination of GMO technologies. In conclusion, this study reaffirms the insufficiency of scientific evidence supporting a causal association between GMOs and human health, while identifying the contextual factors underlying limited signals and the methodological constraints of existing research. At the same time, it underscores the need for societal and international efforts to bridge the divide between public perception, policy, and scientific consensus. The quantitative analyses and structural review presented here provide a balanced understanding of GMOs at the intersection of science and society, and may serve as a critical foundation for future policy development and research agendas. Although the results of this study do not provide clear evidence that the authorization of GMOs exerts a universal and immediate negative effect on the prevalence of major chronic diseases, they nevertheless yield several important policy implications for the assessment and design of GMO-related public health policies. In addition, time-dependent confounding factors – such as changes in screening intensity, cancer registry completeness, and diagnostic criteria over time – may substantially influence the timing and direction of identified breakpoints, independent of GMO authorization. First, the public health associations of GMO policies cannot be adequately evaluated by considering the effects of GMOs alone. Socioeconomic and contextual factors – such as national economic status, population size, dietary patterns, lifestyle behaviors, and the consumption of ultra-processed foods – play a critical role in shaping disease prevalence and should therefore be evaluated in conjunction with GMO authorization. This finding underscores the need to assess GMO policies within a multifactorial public health framework rather than through a single-risk-factor approach. Second, the results suggest that the uniform application of GMO cultivation and distribution policies across countries may obscure important national- and disease-specific differences. The associations between GMO authorization levels and disease prevalence were found to vary according to country-specific characteristics and disease types, indicating that GMO policies should be tailored strategically to national contexts rather than applied in a one-size-fits-all manner. Third, previous ecological analyses have suggested that, for certain diseases (e.g., hypertension, breast cancer, and colorectal cancer), disease prevalence may vary with broader agricultural and dietary contexts, including the scale of GMO cultivation; however, such associations remain inconsistent and non-causal. This finding highlights the importance of rigorous pre- and post-implementation monitoring when introducing or expanding GMO-related policies. In this regard, GMO authorization should be treated not as a one-time regulatory decision but as an adaptive policy process accompanied by long-term and systematic health association monitoring. Taken together, these findings indicate that GMO authorization policies should be understood not merely as decisions regarding technological adoption, but as integrated policy domains encompassing public health, food systems, and socioeconomic environments. Future policy development should therefore incorporate disease prevention strategies aligned with national demographic and dietary characteristics, alongside continuous health monitoring and lifestyle-related public health interventions, to ensure evidence-based and context-sensitive GMO governance.

## Nomenclature

GMO: Genetically Modified Organism
HT: Herbicide-Tolerant
IR: Insect-Resistant
WHO: World Health Organization
EFSA: European Food Safety Authority
NAS: National Academy of Sciences
GBD: Global Burden of Disease Study
GLOBOCAN: Global Cancer Observatory
CDC: Centers for Disease Control and Prevention
ECDC: European Center for Disease Prevention and Control

## Supplementary Material

Supplementary Table.docx

## Data Availability

All data used in this study are publicly available from international databases. Cancer incidence and mortality data were obtained from the Global Burden of Disease Study (GBD; http://ghdx.healthdata.org/gbd-results-tool.) and the Global Cancer Observatory (GLOBOCAN; https://gco.iarc.fr/). Additional epidemiological data were retrieved from the World Health Organization (WHO; https://www.who.int/data/.), the Centers for Disease Control and Prevention (CDC WONDER; https://wonder.cdc.gov/.), and the European Centre for Disease Prevention and Control (ECDC; https://www.ecdc.europa.eu/). Extracted datasets and analytic code (Joinpoint regression outputs) are available from the corresponding author upon reasonable request.
