# Supplementary material for: Genetically modified foods and human health: a comprehensive review and cross-national time-trend analysis
Source: GM Crops Food. 2026 Mar 2;17(1):2634489. doi: 10.1080/21645698.2026.2634489 (PMC12959178; doi:10.1080/21645698.2026.2634489)
Supplement: Supplementary Table.docx [file KGMC_A_2634489_SM1989.docx]

Supplementary Table 1. List for cancer types in analyses

| No | Cancer type | Association with GMO | Epidemiological Evidence and Mechanistics | Reference |
| --- | --- | --- | --- | --- |
| 1 | Neoplasms | Unknown | Lack of evidence on general neoplasm risk and GMO exposure | (Kuiper et al. 2001) |
| 2 | Esophageal cancer | No | No epidemiologic link between GMO exposure and esophageal cancer | (Wang et al. 2014, Bawa and Anilakumar 2013) |
| 3 | Stomach cancer | No | No change in stomach cancer incidence related to GMO exposure | (Zheng et al. 2014), (Cancer 2015) |
| 4 | Liver cancer | No | No epidemiologic evidence linking GMO exposure and liver cancer | (Zheng et al. 2014), (Sun et al. 2025) |
| 5 | Larynx cancer | No | No reported epidemiological link between GMO and larynx cancer | (Cui et al. 2009) |
| 6 | Tracheal, bronchus, and lung cancer | No | No association found between GMO consumption and lung cancer | (Molina et al. 2008)(Zou et al. 2022)(Reid, Santella, and Ambrosone 2008) |
| 7 | Breast cancer | Inconclusive | Insufficient epidemiologic evidence; potential endocrine disruption | (Eve et al. 2020(Calaf et al. 2020) |
| 8 | Cervical cancer | No | Primarily HPV-related; no GMO-related association reported | (Calaf et al. 2020) |
| 9 | Uterine cancer | No | No epidemiologic evidence linking GMO exposure to uterine cancer | (Cancer 2015) |
| 10 | Prostate cancer | Inconclusive | No clear epidemiologic link; possible endocrine disruption | (Alrawi and Al-Rawi 2022) |
| 11 | Colon and rectum cancer | Inconclusive | Possible mechanism via gut microbiome alteration; insufficient epidemiologic evidence | (Song, Chan, and Sun 2020)(Sánchez-Alcoholado et al. 2020)(Colombo et al. 2022) |
| 12 | Lip and oral cavity cancer | No | No epidemiological association with GMO exposure | (Kuiper et al. 2001)(Minot et al. 2024) |
| 13 | Nasopharynx cancer | No | Primarily EBV-related; no link with GMO exposure | (Huang et al. 2017) |
| 14 | Other pharynx cancer | No | No epidemiologic link to GMO exposure | (Cancer 2015) |
| 15 | Gallbladder and biliary tract cancer | No | No epidemiologic evidence linking GMO exposure | (Zheng et al. 2014) |
| 16 | Pancreatic cancer | No | No reported association with GMO exposure | (Alrawi and Al-Rawi 2022) |
| 17 | Malignant skin melanoma | No | No association with GMO exposure | (Cancer 2015) |
| 18 | Non-melanoma skin cancer | No | No association with GMO exposure | (Kuiper et al. 2001) |
| 19 | Ovarian cancer | No | No evidence linking GMO exposure and ovarian cancer | (Cancer 2015) |
| 20 | Testicular cancer | No | No reported link with GMO exposure | (Zheng et al. 2014) |
| 21 | Kidney cancer | No | No epidemiologic evidence linking GMO exposure to kidney cancer | (Cancer 2015) |
| 22 | Bladder cancer | No | No epidemiologic evidence of a link with GMO exposure | (Kuiper et al. 2001) |
| 23 | Brain and central nervous system cancer | No | No epidemiologic evidence linking GMO exposure to CNS cancers | (Alrawi and Al-Rawi 2022) |
| 24 | Thyroid cancer | No | No association with GMO exposure | (Sandler et al. 2018) |
| 25 | Mesothelioma | No | Asbestos-related; no association with GMO | (Farahmand et al. 2023)(Both, Turner, and Henderson 1995) |
| 26 | Hodgkin lymphoma | No | No epidemiologic evidence linking GMO to Hodgkin lymphoma | (Chang and Delzell 2016) |
| 27 | Non-hodgkin lymphoma | Limited | Epidemiologic link with glyphosate exposure demonstrated | (Boffetta et al. 2021) |
| 28 | Multiple myeloma | Limited | Increased risk in agricultural workers exposed to glyphosate | (Chang and Delzell 2016)(Sorahan 2015) |
| 29 | Leukemia | Inconclusive | Limited possible association with glyphosate exposure reported | (Chang and Delzell 2016)(Ward et al. 2023) |
| 30 | Other malignant neoplasms | Inconclusive | Insufficient epidemiologic evidence linking GMO | (Kuiper et al. 2001) |
| 31 | Other neoplasms | Inconclusive | Limited epidemiologic research related to GMO | (Alrawi and Al-Rawi 2022) |
| 32 | Eye cancer | No | No association with GMO exposure | (Cancer 2015) |
| 33 | Soft tissue and other extraosseous sarcomas | No | No association with GMO exposure | (Chan et al. 2017) |
| 34 | Malignant neoplasm of bone and articular cartilage | No | No epidemiologic evidence linking GMO exposure | (Chan et al. 2017) |
| 35 | Neuroblastoma and other peripheral nervous cell tumors | No | No reported association with GMO exposure | (Kuiper et al. 2001) |

Kuiper, Harry A, Gijs A Kleter, Hub PJM Noteborn, and Esther J Kok. 2001. 'Assessment of the food safety issues related to genetically modified foods', *The plant journal, 27: 503-28.*

Wang, An-Hui, Yuan Liu, Bo Wang, Yi-Xuan He, Ye-Xian Fang, and Yong-Ping Yan. 2014. 'Epidemiological studies of esophageal cancer in the era of genome-wide association studies', *World Journal of Gastrointestinal Pathophysiology, 5: 335.*

Bawa, AS, and KR Anilakumar. 2013. 'Genetically modified foods: safety, risks and public concerns—a review', *Journal of food science and technology, 50: 1035-46.*

Zheng, Weiling, Zhen Li, Anh Tuan Nguyen, Caixia Li, Alexander Emelyanov, and Zhiyuan Gong. 2014. 'Xmrk, kras and myc transgenic zebrafish liver cancer models share molecular signatures with subsets of human hepatocellular carcinoma', *PLoS One, 9: e91179.*

Cancer, International Agency for Research on. 2015. "IARC Monograph on Glyphosate." In.: Who.

Sun, Liangchao, Kaikai Zhao, Xiaoli Liu, and Xue Meng. 2025. 'Global, regional, and national burden of esophageal cancer using the 2019 global burden of disease study', *Scientific Reports, 15: 3284.*

Cui, Ri, Koichi Matsuda, Yoichiro Kamatani, and Yusuke Nakamura. 2009. 'Abstract# 2109: Gene-gene and gene-environmental interactions enhance risks of esophageal squamous cell cancer', *Cancer Research, 69: 2109-09.*

Molina, Julian R, Ping Yang, Stephen D Cassivi, Steven E Schild, and Alex A Adjei. 2008. "Non-small cell lung cancer: epidemiology, risk factors, treatment, and survivorship." In *Mayo clinic proceedings, 584-94. Elsevier.*

Zou, Kaiyong, Peiyuan Sun, Huang Huang, Haoran Zhuo, Ranran Qie, Yuting Xie, Jiajun Luo, Ni Li, Jiang Li, and Jie He. 2022. 'Etiology of lung cancer: Evidence from epidemiologic studies', *Journal of the National Cancer Center, 2: 216-25.*

Reid, Mary E, Regina Santella, and Christine B Ambrosone. 2008. 'Molecular epidemiology to better predict lung cancer risk', *Clinical lung cancer, 9: 149-53.*

Eve, Louisane, Béatrice Fervers, Muriel Le Romancer, and Nelly Etienne-Selloum. 2020. 'Exposure to endocrine disrupting chemicals and risk of breast cancer', *International journal of molecular sciences, 21: 9139.*

Calaf, Gloria M, Richard Ponce-Cusi, Francisco Aguayo, Juan P Muñoz, and Tammy C Bleak. 2020. 'Endocrine disruptors from the environment affecting breast cancer', *Oncology letters, 20: 19-32.*

Alrawi, Rakhad Abdulrazak, and Rafal Abdulrazak Al-Rawi. 2022. 'Facts and horizons of genetically modified organisms/foods and health issues', *World Journal of Advanced Research and Reviews, 2: 071-75.*

Song, Mingyang, Andrew T Chan, and Jun Sun. 2020. 'Influence of the gut microbiome, diet, and environment on risk of colorectal cancer', *Gastroenterology, 158: 322-40.*

Sánchez-Alcoholado, Lidia, Bruno Ramos-Molina, Ana Otero, Aurora Laborda-Illanes, Rafael Ordóñez, José Antonio Medina, Jaime Gómez-Millán, and María Isabel Queipo-Ortuño. 2020. 'The role of the gut microbiome in colorectal cancer development and therapy response', *Cancers, 12: 1406.*

Colombo, Francesca, Oscar Illescas, Sara Noci, Francesca Minnai, Giulia Pintarelli, Angela Pettinicchio, Alberto Vannelli, Luca Sorrentino, Luigi Battaglia, and Maurizio Cosimelli. 2022. 'Gut microbiota composition in colorectal cancer patients is genetically regulated', *Scientific Reports, 12: 11424.*

Kuiper, Harry A, Gijs A Kleter, Hub PJM Noteborn, and Esther J Kok. 2001. 'Assessment of the food safety issues related to genetically modified foods', *The plant journal, 27: 503-28.*

Minot, Samuel S, Naisi Li, Harini Srinivasan, Jessica L Ayers, Ming Yu, Sean T Koester, Mary M Stangis, Jason A Dominitz, Richard B Halberg, and William M Grady. 2024. 'Colorectal cancer-associated bacteria are broadly distributed in global microbiomes and drivers of precancerous change', *Scientific Reports, 14: 23646.*

Huang, DI, Shi-Jian Song, Zi-Zhao Wu, Wei Wu, Xiu-Ying Cui, Jia-Ning Chen, Mu-Sheng Zeng, and Shi-Cheng Su. 2017. 'Epstein–Barr virus-induced VEGF and GM-CSF drive nasopharyngeal carcinoma metastasis via recruitment and activation of macrophages', *Cancer Research, 77: 3591-604.*

Sandler, Jason E, Huang Huang, Nan Zhao, Weiwei Wu, Fangfang Liu, Shuangge Ma, Robert Udelsman, and Yawei Zhang. 2018. 'Germline variants in DNA repair genes, diagnostic radiation, and risk of thyroid cancer', *Cancer Epidemiology, Biomarkers & Prevention, 27: 285-94.*

Farahmand, Pooyeh, Katarina Gyuraszova, Claire Rooney, Ximena L Raffo-Iraolagoitia, Geeshath Jayasekera, Ann Hedley, Emma Johnson, Tatyana Chernova, Gaurav Malviya, and Holly Hall. 2023. 'Asbestos accelerates disease onset in a genetic model of malignant pleural mesothelioma', *Frontiers in Toxicology, 5: 1200650.*

Both, Katrin, David R Turner, and Douglas W Henderson. 1995. 'Loss of heterozygosity in asbestos‐induced mutations in a human mesothelioma cell line', *Environmental and molecular mutagenesis, 26: 67-71.*

Chang, Ellen T, and Elizabeth Delzell. 2016. 'Systematic review and meta-analysis of glyphosate exposure and risk of lymphohematopoietic cancers', *Journal of Environmental Science and Health, Part B, 51: 402-34.*

Boffetta, Paolo, Catalina Ciocan, Carlotta Zunarelli, and Enrico Pira. 2021. 'Exposure to glyphosate and risk of non-Hodgkin lymphoma: an updated meta-analysis', *La Medicina del lavoro, 112: 194.*

Sorahan, Tom. 2015. 'Multiple myeloma and glyphosate use: a re-analysis of US Agricultural Health Study (AHS) data', *International Journal of Environmental Research and Public Health, 12: 1548-59.*

Ward, Mary H, Jessica M Madrigal, Rena R Jones, Melissa C Friesen, Roni T Falk, David Koebel, and Catherine Metayer. 2023. 'Glyphosate in house dust and risk of childhood acute lymphoblastic leukemia in California', *Environment international, 172: 107777.*

Chan, Sock Hoai, Weng Khong Lim, Nur Diana Binte Ishak, Shao-Tzu Li, Wei Lin Goh, Gek San Tan, Kiat Hon Lim, Melissa Teo, Cedric Ng Chuan Young, and Simeen Malik. 2017. 'Germline mutations in cancer predisposition genes are frequent in sporadic sarcomas', *Scientific Reports, 7: 10660.*
